# Large dentigerous cyst associated with the maxillary impacted supernumerary teeth: A rare occurrence and literature review

**DOI:** 10.34172/joddd.2022.043

**Published:** 2022-12-30

**Authors:** Noriaki Aoki, Megumi Matumoto, Soichiro Ishii, Yasuaki Okuma, Himiko Umezawa, Junichi Baba, Takaaki Ito

**Affiliations:** ^1^Department of Oral and Maxillofacial Surgery, Saiseikai Yokohamashi Nanbu Hospital, Japan; ^2^Department of Oral and Maxillofacial Surgery, Shirayuri Beauty Clinic, Japan

**Keywords:** Dentigerous cyst, Supernumerary tooth, Case report

## Abstract

Dentigerous cysts are common odontogenic cysts of the jaw but are rarely associated with supernumerary teeth. Few cases of large dentigerous cysts associated with anterior maxillary supernumerary teeth have been reported. The English literature has documented only four cases of dentigerous cysts>40 mm in diameter associated with supernumerary teeth. A 47-year-old man was referred to our hospital, complaining of minor pain in the maxillary gingiva. Computed tomography revealed a well-defined oval unilocular radiolucent lesion (50×45×35 mm) in the right maxilla, including two impacted supernumerary teeth. A dentigerous cyst associated with impacted anterior maxillary supernumerary teeth was diagnosed. The two impacted teeth were surgically removed, and the cyst was enucleated using the Caldwell-Luc approach. Histopathology confirmed the diagnosis of a large dentigerous cyst associated with impacted anterior maxillary supernumerary teeth. The postoperative course has been uneventful for two years. We also reviewed the relevant English literature.

## Introduction

Dentigerous cysts are common odontogenic cysts of the jaw, associated with unerupted teeth, usually impacted mandibular third molars, maxillary canines, and mandibular premolars. Dentigerous cysts associated with anterior supernumerary teeth are rare, accounting for 5–6% of dentigerous cysts.^1,2^ Dentigerous cysts occur due to the separation of the dental germ around the crown of an unerupted tooth.^3^ There is a slight predominance of the condition in males.^2,4^ Dentigerous cysts are often discovered during routine radiographic examinations in clinical dental practice and are usually removed to allow the involved tooth to develop.

A search of PubMed from 1970 to 2022 using the key words “dentigerous cyst”, “maxillary supernumerary tooth”, and “maxillary sinus” was conducted. To our knowledge, only four cases of dentigerous cysts > 40 mm in diameter associated with supernumerary teeth have been documented in the English literature (Table 1).^5-8^ This article describes an extremely rare case of a large dentigerous cyst associated with impacted anterior maxillary supernumerary teeth and reviews the literature.

**Table 1:** Dentigerous cyst with anterior maxillary supernumerary teeth, more than 40mm in size as reported in the English literatures

**Reported year**	**First Author**	**Gender/Age**	**Main symptom**	**Site**	**Size (mm)**	**Duration**	**Preoperative biopsy**	**Treatment**	**Bone graft **	**Approach**
1982	Most^5^	M/30	Swelling	Right	90 x 15 x 80	Unknown	None	Enucleation	None	Vestibule incision
2012	Ngamdu^6^	M/18	Buccal swelling	Right	80 x 60	2 years	None	Enucleation	None	Caldwell -Luc
2013	Kim^7^	M/35	Painful swelling	Left	45 x 25 x 24	3 weeks	None	Enucleation	None	Caldwell -Luc
2014	Navarro^8^	M/23	Slight swelling	Both sites	60 x 30	2 months	None	Enucleation	Xenograft	Cervical incision from 13 to 23
2022	Present case	M/48	Slight pain	Right	50 x 45 x 35	2 years	Cytology, histopathology	Enucleation	None	Caldwell -Luc

## Case Report

**Figure 1:**
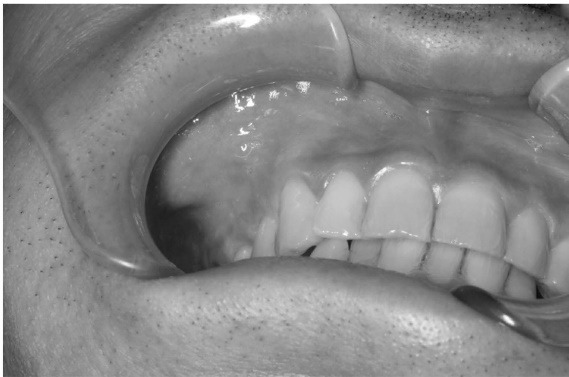
Intraoral photograph in the initial visit showing swelling in the vestibule

**Figure 2:**
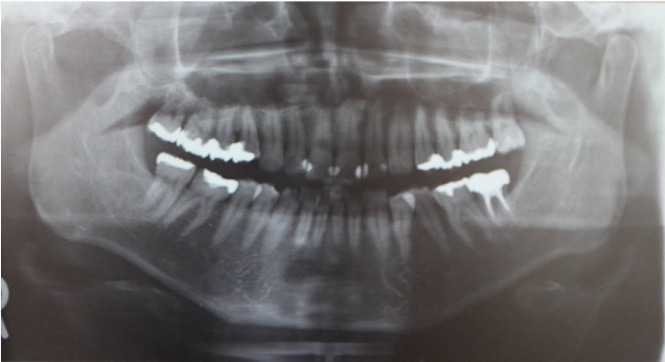
The panoramic radiograph showing a large radiolucent area (white arrowhead), including two radiopaque tooth-like structures (white arrow)

**Figure 3:**
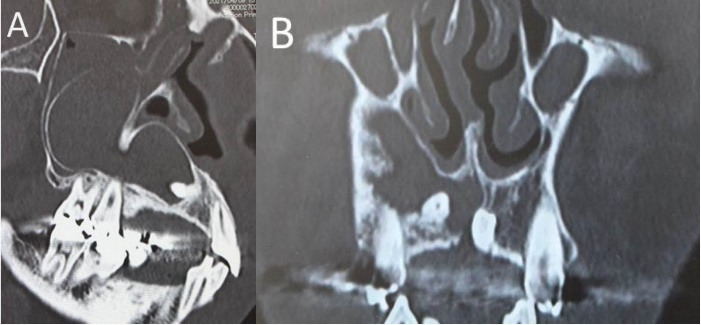
Computed tomography image. A: Sagittal image showing a 50 × 45 × 35-mm cystic lesion B: Coronal image showing two supernumerary teeth

A 47-year-old man visited our hospital with a chief complaint of discomfort and slight intermittent pain in the maxillary gingiva. He had noticed a diffuse swelling in the anterior maxillary area over the past 6 months. On oral examination, a 30 × 25-mm solitary well-defined swelling was seen in the maxillary labial vestibule, extending from the right central incisor to the first molar. The swelling was tender and fluctuant on palpation (Figure 1). The right maxillary teeth were not mobile and exhibited a vital pulp reaction. The panoramic radiograph revealed a large radiolucent area close to the roots of teeth #12 to #16, extending to the right orbital rim, including two radiopaque structures resembling teeth (Figure 2). A computed tomographic (CT) examination revealed an oval unilocular radiolucent lesion, approximately 50 × 45 × 35 mm in size with a well-defined border in the right maxilla and including two supernumerary teeth (Figure 3A, B).

**Figure 4:**
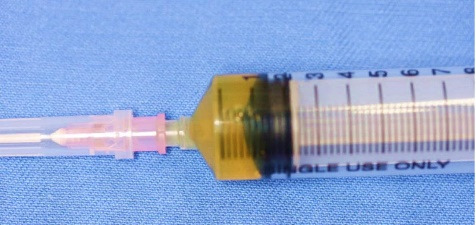
Contents from the puncture showing 1.5 mL of yellowish cystic fluid

**Figure 5:**
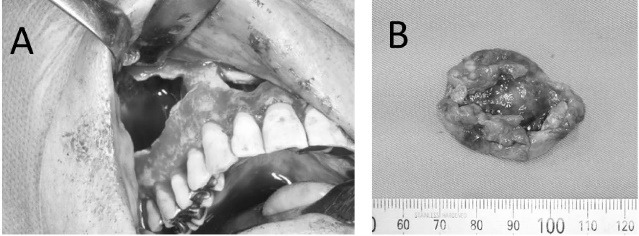
Intraoperative photograph. A: Cyst enucleation and tooth extraction performed. B: The cyst with supernumerary teeth (black arrow)

**Figure 6:**
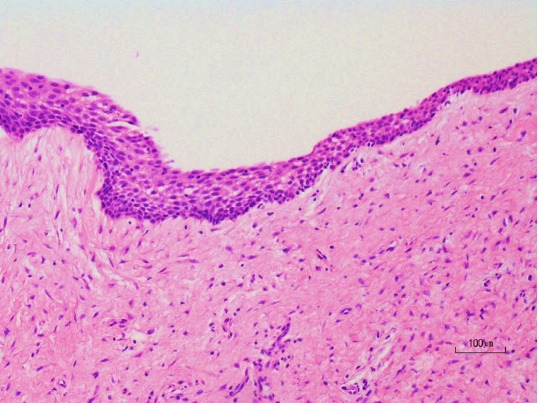
Hematoxylin-eosin staining. Cyst wall lined by nonkeratinized stratified squamous epithelium ( × 20)

The maxillary sinus was mostly occupied by this lesion which elevated the sinus floor; however, the original sinus was confirmed inside the maxilla. Hypertrophy of the maxillary sinus mucosa was found. Cytology confirmed it was a Class I lesion, while fine needle aspiration (FNA) yielded 1.5 mL of yellowish cystic fluid contents with cholesterin crystals (Figure 4). A mucoperiosteal flap was reflected, the thin buccal bone was removed, and a specimen was taken. Histopathological biopsy revealed that the odontogenic cyst wall was lined with *stratified squamous epithelium*. Our clinical diagnosis was a dentigerous cyst associated with impacted anterior maxillary supernumerary teeth. Surgical removal of the two impacted supernumerary teeth and enucleation of the cyst were simultaneously performed using the Caldwell-Luc approach (Figure 5A, B). No evidence was found of sinus mucosa in the cyst cavity. The two supernumerary teeth, which had adhesions to the cyst wall, were surgically removed. Intraoperatively, the lesion appeared to abut against the palatal bone, but no adhesion was found between the palatal bone and cyst wall. Finally, the buccal mucoperiosteal flap was repositioned and closed with resorbable sutures as the primary closure. Hematoxylin-eosin staining showed that the lumen of the cyst wall was lined by 5–6 layers of nonkeratinized stratified squamous epithelium (Figure 6). The final histopathological diagnosis was a dentigerous cyst. The postoperative course has been uneventful for two years.

## Discussion

Dentigerous cysts are common odontogenic jaw cysts and are associated with unerupted teeth. However, dentigerous cysts with supernumerary teeth are rarely found and account for only 5% of all dentigerous cysts. To our knowledge, dentigerous cysts measuring > 40 mm in diameter occur very rarely, with only four cases reported in the English literature.^5-8^

Dentigerous cysts may appear radiographically as well-defined unilocular or multilocular radiolucent lesions, including the crown of an unerupted tooth.^7^ Images of supernumerary teeth are clearly seen radiographically. Panoramic radiographs, plain radiographs of the maxillary sinus using Waters’ method, and CT, in particular, are useful for determining the location of the tooth. CT imaging is the gold standard modality for confirming the location of the unerupted tooth and the spread of the cyst.^1,2^ In the present case, panoramic radiography and CT were performed to diagnose the intra-sinus situation. The relationship between the original sinus and the cystic cavity was not obvious on the panoramic radiograph because the vertebra overlapped the sinus structure. Since a thin septum was evident between the original sinus and the cystic cavity on CT, we could confirm that the lesion existed within the maxillary bone, separate from the sinus.

The differential diagnosis of dentigerous cysts includes ameloblastoma, odontogenic keratocyst, odontogenic fibroma, radicular cyst, residual cyst, odontogenic myxoma, adenomatoid odontogenic tumor, odontoma, and Pindborg tumor,^9-14^ all of which may have a similar radiographic appearance. Occasionally, a dentigerous cyst must be differentiated from a retention cyst of the maxillary sinus mucosa when maxillary sinus mucosa hypertrophy is involved.^10^ Malignant tumors must be ruled out because dentigerous cysts can develop into other tumor types, including squamous cell carcinoma and mucoepidermoid carcinoma.^11,13^ A rare case of an adenomatoid odontogenic tumor associated with impacted supernumerary teeth was reported by Mohanty et al.^11^ It is difficult to diagnose a dentigerous cyst at the initial visit. Therefore, a histopathological examination is necessary to reach a definitive diagnosis because the clinical diagnosis may be confused with another type of odontogenic tumor. Early detection and treatment are essential because of the potential of dentigerous cysts to develop into large odontogenic tumors such as ameloblastomas and malignant tumors such as squamous cell carcinoma and mucoepidermoid carcinoma.^10^ In the present case, the lesion was a painless swelling that had already developed to a large size in the maxillary sinus. A biopsy was performed to establish the definitive diagnosis after the cytology revealed it was a Class I lesion.

Avitia et al^14^ reported a case of a large dentigerous cyst that led to orbital proptosis and exophthalmos because of the rapid growth of a lesion in the maxillary sinus, measuring 5 cm in diameter. Altas et al^12^ documented a large dentigerous cyst containing a canine and involving the entire maxillary sinus, resulting in epiphora. Nasal obstruction and epiphora were caused by cystic pressure affecting the medial wall of the sinus and the nasolacrimal canal. These findings are uncommon in dentigerous cysts. Ray et al^15^ reported a case of a dentigerous cyst with a supernumerary tooth in the sinus of an 11-year-old male child, obstructing the nasolacrimal duct. Such clinical reports of nasolacrimal duct obstruction from developing dentigerous cysts in the sinus are extremely rare in the literature.^3^ Early diagnosis and appropriate treatment planning are crucial in such uncommon cases to avoid further complications.

The standard treatments for dentigerous cysts are primary closure after cyst enucleation, open wound after cyst enucleation, and marsupialization while retaining the cyst wall. Treatment with an open wound after cyst enucleation promotes bone regeneration from the wall of the cystic cavity, leading to the shrinkage of the cyst. These treatment methods each have several advantages and disadvantages.^3^ For example, although marsupialization results in less surgical stress and possible avoidance of removal of the involved tooth, the treatment is prolonged and requires the gauze inserted in the cavity to be irrigated every 2–3 weeks in the clinic to prevent the closure of the marsupialization hole. The treatment duration for primary closure is short; however, there is a risk of infection and poor healing postoperatively because of the large dead space left after closure. Generally, primary closure is selected for small cysts, while an open wound and marsupialization may be more suitable for large cysts. Thus, we could have selected open wound treatment or marsupialization for the present case. However, primary closure was selected because the patient refused to undergo prolonged treatment, and it was thought that it could be successfully managed without complications. All the previous reports of large dentigerous cysts with a diameter of > 40 mm associated with the impacted maxillary supernumerary teeth were treated with cyst enucleation (Table 1).^5-8^

Controversy surrounds the management of the large residual bone cavity and whether it should be filled with a bone graft. Scolozzi et al^16^ described the enucleation of a cyst followed by a bone graft procedure from the iliac bone. Richter et al^17^ also recommended simultaneous cyst enucleation and bone grafts for large cysts. In contrast, healing and spontaneous bone formation in the cavity were observed 6‒24 months after surgery in studies by Wagdargi and Chiapasco.^18,19^ Many studies have reported filling large bone cavities of the jaw with bone material after cyst enucleation.^17,20^ Numerous graft materials are available, including autogenous grafts, allogenic grafts, xenografts, and platelet-rich plasma, which also possess excellent space-maintaining properties because the high concentration of osteoinductive cells provides an osteoconductive scaffold during bone formation. Bodner^20^ reported the effectiveness of a decalcified freeze-dried bone allograft on the healing of a 50-mm dentigerous cyst after cyst enucleation,^17,20^ demonstrating that allografts promote bone healing after cyst removal. In the present case, surgical removal of the impacted supernumerary teeth and cyst enucleation were performed without a bone graft. The surgical bone defect of the cystic cavity was 5 × 3 cm; however, no bone graft was used to assist spontaneous bone regeneration. In our review of the literature, we found only four cases of cysts measuring > 40 mm associated with supernumerary teeth, and three of these were treated without bone grafts (Table 1).^5-8^

## Conclusion

 This case report described an extremely rare case of a large dentigerous cyst associated with impacted maxillary supernumerary teeth and reviewed the English literature. The information provided will contribute to successful diagnosis and choice of treatment modality when clinicians encounter a large dentigerous cyst in routine dental practice.

## Funding

 None.

## Ethics Approval

 Consent for publishing photographs was obtained from the patient.

## Competing Interests

 The authors declare no conflicts of interest relevant to this article.

## References

[1] Shah KM, Karagir A, Adaki S, Pattanshetti C. Dentigerous cyst associated with an impacted anterior maxillary supernumerary tooth. BMJ Case Rep 2013;2013. 10.1136/bcr-2012-008329. PMC3604207 23376673

[2] Agrawal NK (2012). Dentigerous cyst in a child associated with multiple inverted supernumerary teeth: a rare occurrence. Int J Burns Trauma.

[3] Kara MI, Yanik S, Altan A, Oznalcin O, Ay S (2015). Large dentigerous cyst in the maxillary sinus leading to diplopia and nasal obstruction: case report. J Istanb Univ Fac Dent.

[4] Hasan S, Ahmed SA, Reddy LB (2014). Dentigerous cyst in association with impacted inverted mesiodens: Report of a rare case with a brief review of literature. Int J Appl Basic Med Res.

[5] Most DS, Roy EP (1982). A large dentigerous cyst associated with a supernumerary tooth. J Oral Maxillofac Surg.

[6] Ngamdu YB, Kodiya AM, Sandabe MB, Garandawa HI, Isa A (2012). Dentigerous cyst associated with ectopic supernumerary canine in the maxillary sinus. J Case Rep.

[7] Kim KS, Mun SK (2013). Extensive dentigerous cyst associated with a mesiodens: CT findings. Ear Nose Throat J.

[8] Navarro BG, Jané Salas E, Olmo IT, AF IM, Juarez Escalona I, López-López J (2014). Maxillary dentigerous cyst and supernumerary tooth. Is it a frequent association? Oral Health Dent Manag.

[9] Wanjari SP, Tekade SA, Parwani RN, Managutti SA (2011). Dentigerous cyst associated with multiple complex composite odontomas. Contemp Clin Dent.

[10] Ustuner E, Fitoz S, Atasoy C, Erden I, Akyar S (2003). Bilateral maxillary dentigerous cysts: a case report. Oral Surg Oral Med Oral Pathol Oral Radiol Endod.

[11] Mohanty R, Singh V, Dey AK, Behera S (2019). A rare nonsyndromic case of adenomatoid odontogenic tumor associated with multiple impacted supernumerary teeth. Natl J Maxillofac Surg.

[12] Altas E, Karasen RM, Yilmaz AB, Aktan B, Kocer I, Erman Z (1997). A case of a large dentigerous cyst containing a canine tooth in the maxillary antrum leading to epiphora. J Laryngol Otol.

[13] Tournas AS, Tewfik MA, Chauvin PJ, Manoukian JJ (2006). Multiple unilateral maxillary dentigerous cysts in a non-syndromic patient: a case report and review of the literature. Int J Pediatr Otorhinolaryngol Extra.

[14] Avitia S, Hamilton JS, Osborne RF (2007). Dentigerous cyst presenting as orbital proptosis. Ear Nose Throat J.

[15] Ray B, Bandyopadhyay SN, Das D, Adhikary B (2009). A rare cause of nasolacrimal duct obstruction: dentigerous cyst in the maxillary sinus. Indian J Ophthalmol.

[16] Scolozzi P, Lombardi T, Richter M (2005). Upper lip swelling caused by a large dentigerous cyst. Eur Arch Otorhinolaryngol.

[17] Richter M, Laurent F, Chausse JM (1986). Homologous cancellous bone grafts for large jaw defects caused by bone cysts. J Oral Maxillofac Surg.

[18] Wagdargi SS, Rai KK, Arunkumar KV, Katkol B, Arakeri G (2016). Evaluation of spontaneous bone regeneration after enucleation of large cysts of the jaws using radiographic computed software. J Contemp Dent Pract.

[19] Chiapasco M, Rossi A, Motta JJ, Crescentini M (2000). Spontaneous bone regeneration after enucleation of large mandibular cysts: a radiographic computed analysis of 27 consecutive cases. J Oral Maxillofac Surg.

[20] Bodner L (1996). Effect of decalcified freeze-dried bone allograft on the healing of jaw defects after cyst enucleation. J Oral Maxillofac Surg.

